# Pathogenic variants in *GCSH* encoding the moonlighting H-protein cause combined nonketotic hyperglycinemia and lipoate deficiency

**DOI:** 10.1093/hmg/ddac246

**Published:** 2022-10-03

**Authors:** Laura Arribas-Carreira, Cristina Dallabona, Michael A Swanson, Joseph Farris, Elsebet Østergaard, Konstantinos Tsiakas, Maja Hempel, Cecile Aquaviva-Bourdain, Stefanos Koutsoukos, Nicholas V Stence, Martina Magistrati, Elaine B Spector, Kathryn Kronquist, Mette Christensen, Helena G Karstensen, René G Feichtinger, Melanie T Achleitner, J Lawrence Merritt II, Belén Pérez, Magdalena Ugarte, Stephanie Grünewald, Anthony R Riela, Natalia Julve, Jean-Baptiste Arnoux, Kasturi Haldar, Claudia Donnini, René Santer, Allan M Lund, Johannes A Mayr, Pilar Rodriguez-Pombo, Johan L K Van Hove

**Affiliations:** Centro de Diagnóstico de Enfermedades Moleculares, Centro de Biología Molecular Severo Ochoa, CBM-CSIC, Departamento de Biología Molecular, Institute for Molecular Biology-IUBM, Universidad Autónoma Madrid, CIBERER, IDIPAZ, 28049 Madrid, Spain; Department of Chemistry, Life Sciences and Environmental Sustainability, University of Parma, Parma, Italy; Department of Pediatrics, Section of Clinical Genetics and Metabolism, University of Colorado, Aurora, CO, USA; Boler-Parseghian Center for Rare and Neglected Disease, and Department of Biological Sciences, University of Notre Dame, Notre Dame, IN, USA; Department of Biology, Saginaw Valley State University, University Center, MI, USA; Centre for Inherited Metabolic Diseases, Departments of Clinical Genetics and Pediatrics, Rigshospitalet - Copenhagen University Hospital, Copenhagen, Denmark; Department of Pediatrics, University Medical Center Hamburg Eppendorf, Hamburg, Germany; Institute of Human Genetics, University Medical Center Hamburg Eppendorf, Hamburg, Germany; Service Biochimie et Biologie Moléculaire, UM Pathologies Héréditaires du Métabolisme et du Globule Rouge, Centre de Biologie Est, CHU de Lyon, Lyon, France; Department of Pediatrics, Section of Clinical Genetics and Metabolism, University of Colorado, Aurora, CO, USA; Department of Radiology, University of Colorado, Aurora, CO, USA; Department of Chemistry, Life Sciences and Environmental Sustainability, University of Parma, Parma, Italy; Department of Pediatrics, Section of Clinical Genetics and Metabolism, University of Colorado, Aurora, CO, USA; Molecular Genetics Lab, Precision DX, Children's Hospital Colorado, Aurora, CO, USA; Department of Pediatrics, Section of Clinical Genetics and Metabolism, University of Colorado, Aurora, CO, USA; Molecular Genetics Lab, Precision DX, Children's Hospital Colorado, Aurora, CO, USA; Centre for Inherited Metabolic Diseases, Departments of Clinical Genetics and Pediatrics, Rigshospitalet - Copenhagen University Hospital, Copenhagen, Denmark; Centre for Inherited Metabolic Diseases, Departments of Clinical Genetics and Pediatrics, Rigshospitalet - Copenhagen University Hospital, Copenhagen, Denmark; University Children’s Hospital, Salzburger Landeskliniken (SALK) and Paracelsus Medical University (PMU), Salzburg, Austria; University Children’s Hospital, Salzburger Landeskliniken (SALK) and Paracelsus Medical University (PMU), Salzburg, Austria; Department of Pediatrics, University of Washington, Seattle, WA, USA; Centro de Diagnóstico de Enfermedades Moleculares, Centro de Biología Molecular Severo Ochoa, CBM-CSIC, Departamento de Biología Molecular, Institute for Molecular Biology-IUBM, Universidad Autónoma Madrid, CIBERER, IDIPAZ, 28049 Madrid, Spain; Centro de Diagnóstico de Enfermedades Moleculares, Centro de Biología Molecular Severo Ochoa, CBM-CSIC, Departamento de Biología Molecular, Institute for Molecular Biology-IUBM, Universidad Autónoma Madrid, CIBERER, IDIPAZ, 28049 Madrid, Spain; Department of Metabolic Medicine, Great Ormond Street Hospital, London, UK; Texas Child Neurology, Plano, TX, USA; Department of Pediatrics, IMED Valencia Hospital, Valencia, Spain; Centre de Reference des Maladies Hereditaires, Necker Enfants Malades, Paris, France; Boler-Parseghian Center for Rare and Neglected Disease, and Department of Biological Sciences, University of Notre Dame, Notre Dame, IN, USA; Department of Chemistry, Life Sciences and Environmental Sustainability, University of Parma, Parma, Italy; Department of Pediatrics, University Medical Center Hamburg Eppendorf, Hamburg, Germany; Centre for Inherited Metabolic Diseases, Departments of Clinical Genetics and Pediatrics, Rigshospitalet - Copenhagen University Hospital, Copenhagen, Denmark; University Children’s Hospital, Salzburger Landeskliniken (SALK) and Paracelsus Medical University (PMU), Salzburg, Austria; Centro de Diagnóstico de Enfermedades Moleculares, Centro de Biología Molecular Severo Ochoa, CBM-CSIC, Departamento de Biología Molecular, Institute for Molecular Biology-IUBM, Universidad Autónoma Madrid, CIBERER, IDIPAZ, 28049 Madrid, Spain; Department of Pediatrics, Section of Clinical Genetics and Metabolism, University of Colorado, Aurora, CO, USA

## Abstract

Maintaining protein lipoylation is vital for cell metabolism. The H-protein encoded by *GCSH* has a dual role in protein lipoylation required for bioenergetic enzymes including pyruvate dehydrogenase and 2-ketoglutarate dehydrogenase, and in the one-carbon metabolism through its involvement in glycine cleavage enzyme system, intersecting two vital roles for cell survival. Here, we report six patients with biallelic pathogenic variants in *GCSH* and a broad clinical spectrum ranging from neonatal fatal glycine encephalopathy to an attenuated phenotype of developmental delay, behavioral problems, limited epilepsy and variable movement problems. The mutational spectrum includes one insertion c.293-2_293–1insT, one deletion c.122_(228 + 1_229–1) del, one duplication of exons 4 and 5, one nonsense variant p.Gln76^*^and four missense p.His57Arg, p.Pro115Leu and p.Thr148Pro and the previously described p.Met1?. Via functional studies in patient’s fibroblasts, molecular modeling, expression analysis in *GCSH* knockdown COS7 cells and yeast, and *in vitro* protein studies, we demonstrate for the first time that most variants identified in our cohort produced a hypomorphic effect on both mitochondrial activities, protein lipoylation and glycine metabolism, causing combined deficiency, whereas some missense variants affect primarily one function only. The clinical features of the patients reflect the impact of the *GCSH* changes on any of the two functions analyzed. Our analysis illustrates the complex interplay of functional and clinical impact when pathogenic variants affect a multifunctional protein involved in two metabolic pathways and emphasizes the value of the functional assays to select the treatment and investigate new personalized options.

## Introduction

Some proteins have the unusual property of having more than one independent function, a property termed ‘protein moonlighting’. Such functions can be unrelated or cover related functions, such as sequential steps in the same pathway. This different function can relate to differential cellular localization, oligomerization, integration in different complexes or interactions with substrates (1,2). When a protein has more than one function, it may not be clear which function or whether both functions are affected in a given disease process and thus contribute to the phenotype (2,3). This can complicate understanding genotype–phenotype correlations (3). One such protein that exhibits moonlighting functions is the H-protein, encoded by the *GCSH* gene. The H-protein is pivotal in the biosynthesis and transfer of the cofactor lipoate to several critical cellular energetics enzymes (4–6). In addition, the H-protein provides the lipoate carrier function in the glycine cleavage enzyme (7), which contributes to the generation of one-carbon charged folates, which is vital for cell function and survival (8,9). Thus, this single protein intersects two essential roles in the mitochondrion: one-carbon metabolism and cellular bioenergetics (10,11). Here, we describe the clinical, genetic and cellular consequences of pathogenic variants of the H-protein at the intersection of two complex roles.

**Figure 1:**
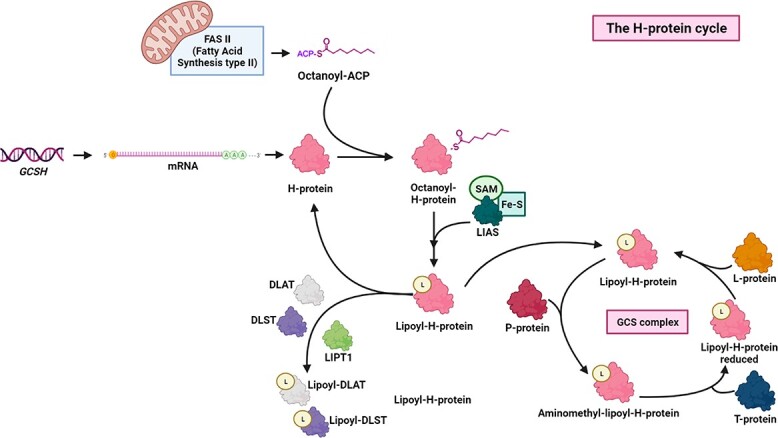
The H-protein cycle. Schematic diagram representing the dual role that the H-protein performs in mitochondria. Once synthesized, the H-protein is central to lipoate synthesis. Following lipoyl-group generation, the H-protein participates in two crucial cellular processes. On one hand, the H-protein donates the lipoyl-group to different bioenergetic enzymes such as DLAT and DLST; on the other hand, the H-protein plays a pivotal role inside the GCS complex which represents the main mechanism of glycine degradation in the cell. ACP: acyl carrier protein; LIAS: lipoyl synthase; SAM: S-adenosylmethionine; LIPT1: lipoyltransferase 1; DLAT: dihydrolipoamide-S-acetyltransferase component of pyruvate dehydrogenase complex; DLST: dihydrolipoamide-S-succinyltransferase component of 2-ketoglutarate dehydrogenase complex; GCS: glycine cleavage enzyme system.

Lipoic acid (6,8-dithio-octanoic acid; lipoate) is an essential, covalently bound co-factor for multiple 2-keto-acid dehydrogenase enzymes, including the key bioenergetic enzymes pyruvate dehydrogenase (PDH) and 2-ketoglutarate dehydrogenase (2-KGDH) (Fig. 1) (5,10). Lipoate biogenesis is initiated by the transfer of octanoate from intramitochondrial fatty acid synthesis (FASII) on octanoyl-ACP through lipoyltransferase 2 (*LIPT2*) onto apo-H protein to generate octanoyl-H (4–6). This octanoyl-H-protein intermediate is then converted to lipoyl-H-protein by a lipoate synthase (*LIAS*)-catalyzed sulfur insertion derived from iron–sulfur clusters and catalyzed by S-adenosylmethionine. Subsequently, lipoyltransferase 1 (*LIPT1*) transfers the lipoyl moiety from H-protein to form an acyl-enzyme intermediate on recipient 2-ketoacid dehydrogenase enzymes such as dihydrolipoamide-S-acetyltransferase (*DLAT*), the E2 component of the PDH complex, and dihydrolipoamide-S-transsuccinylase (*DLST*), the E2 component of the mitochondrial 2-KGDH complex. Lipoate is also a critical cofactor of the glycine cleavage enzyme system (GCS), where the H-protein itself is the lipoate carrier of this enzyme complex. The GCS is the primary system involved in the breakdown of the amino acid glycine. In eukaryotes, GCS consists of four subunits named P-, H-, T- and L- proteins mainly expressed in the brain, liver, kidney and placenta (7). Glycine is decarboxylated to carbon dioxide and the resulting aminomethyl-group transferred to lipoyl-H-protein as aminomethyl-lipoate. The T-protein releases ammonia while transferring the methyl-group to tetrahydrofolate. The L-protein reoxidizes the reduced lipoyl-group. Thus, the H-protein is involved in two intersecting pathways of lipoate biogenesis and transfer reactions, and in glycine catabolism generating 5,10-methylene-tetrahydrofolate (10,11).

Clinically, deficient GCS activity causes nonketotic hyperglycinemia (NKH) (MIM #605899) an autosomal-recessive disorder, characterized biochemically by an accumulation of glycine in body fluids including in cerebrospinal fluid (CSF) with an elevated CSF/plasma glycine ratio (12,13). NKH causes an epileptic encephalopathy which reflects the role of glycine in the central nervous system (14). Patients with severe classic NKH present in the first days of life with an apneic epileptic encephalopathy, which devolves into coma and early demise, or recovery with development of intractable epilepsy, central hypotonia and peripheral spasticity, cortical blindness and absent psychomotor development (13,15,16). Patients with an attenuated presentation of classic NKH, caused by variants preserving some residual GCS function, can present similarly neonatally or have later onset, and make variable developmental progress, have treatable or absent epilepsy, limited or no spasticity, chorea, behavioral problems and hyperactivity (15–17). Nearly 80% of classical NKH patients carry biallelic variants in *GLDC*, encoding the P-protein, with the remaining 20% caused by mutations in the *AMT* gene, encoding the T-protein (18). About 4% of patients with apparent NKH lack variants in *AMT* or *GLDC*. Such ‘variant NKH’ is triggered by deficient lipoylation of proteins including the GCS, but also DLAT and DLST, and associated combined deficiency of PDH and 2-KGDH in addition to the GCS, such as caused by biallelic variants in *LIAS*, *BOLA3*, *NFU1* or *GLRX5* (12,19). Clinical presentations can generally be grouped into neonatal overwhelming disease, early childhood onset disease with multisystem or rapid neurodegenerative course including vacuolating leukodystrophy, or late-onset slowly progressive neurodegenerative course (12).

Three consanguineous patients with the same variant in *GCSH* and without functional or structural analysis to prove pathogenicity (20) were previously reported. Here we report six new patients who presented with NKH and were found to carry bi-allelic variants in *GCSH*. We now expand the clinical phenotypes with this new cohort of non-related patients and perform extensive functional analyses that support pathogenic molecular and biochemical mechanisms and a functional genotype–phenotype correlation.

## Results

### Clinical presentation: case reports

**Figure 2:**
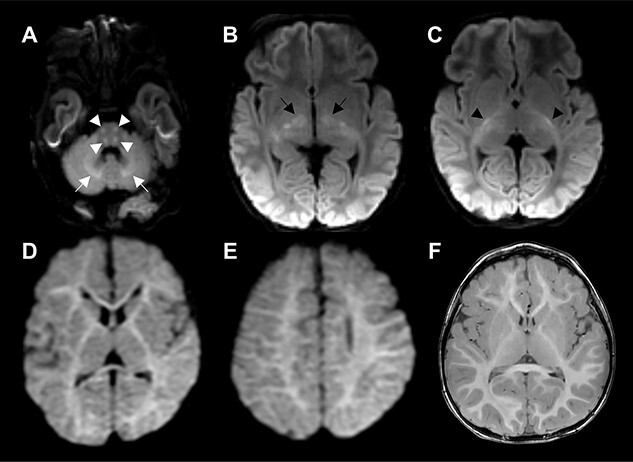
Brain magnetic resonance imaging findings in patients with *GCSH* variants. (**A–C**) Axial diffusion weighted imaging of patient 2 demonstrates areas of diffusion restriction typical of NKH in the brainstem (A, white arrowheads), cerebellar white matter (A, white arrows) and posterior limb internal capsule (C, black arrowheads). Symmetric areas of diffusion restriction in the anterior medial thalami (B, black arrows) are not typical of NKH and may indicate additional cytotoxic edema secondary to PDH deficiency. (**D, E**) Axial diffusion weighted imaging of patient 6 demonstrates a pattern of widespread mildly restricted diffusion throughout the cerebral white matter typical of slightly later stage involvement of NKH. (**F**) Axial T1 imaging of patient 5 demonstrates subjectively mildly low caudate head volume.

A summary of the clinical and laboratory findings of the six unrelated patients included in this study is shown in Table 1. The neuroimaging findings are shown in Figure 2, and detailed case descriptions are available in Supplementary information. Glycine levels in CSF and plasma were all compatible with an NKH diagnosis.

**Table 1:** Clinical and laboratory findings of six patients with variants in *GCSH*

	Patient 1	Patient 2	Patient 3	Patient 4	Patient 5	Patient 6
Origin	Spain	Denmark	USA	UK-USA	Germany	France
Consanguinity	No	No	No	Yes	Yes	Yes
Sex	Female	Male	Female	Female	Male	Male
Pregnancy	Unremarkable	Unremarkable	Hyperemesis (HP:0012188)	Unremarkable	Unremarkable	Unremarkable
Symptom onset	Day 1	Day 3	2 months	6 months	4 days	3 months
Initial symptoms	Lethargy (HP:0001254), hypotonia (HP:0001252)	Lethargy (HP:0001254), hypotonia (HP:0001252) hypoglycemia (HP:0001943)	Exaggerated startles (HP:0002267), infantile spasms (HP:0012469), partial seizures (HP:0007359)	Loss of skills following immunization (trigger by immunization HP:0025219), hypotonia (HP:0001252), developmental delays (HP:0001263)	Sick, apneic (HP:0002104), pallor (HP:0000980) and cyanosis (HP:0000961), requiring intubation and ventilation for 2 weeks (HP:0004887)	Left partial seizures
Main symptoms	Coma, Apnea (HP:0002104)	Coma, hiccups (HP:0100247), hypotonia (HP:0001252), no reflexes (HP:0001284), myoclonias (HP:0003794), ventilation since day 4 (HP:0005946)	Epilepsy (HP:0001250), progressive developmental delay (HP:0001263)	Developmental delays (HP:0001263), hyperactivity (HP:0000752), impulsivity (HP:0100710), rare hallucinations (HP:0000738), limited communication	Since age 3 months: developmental delays (HP:0001263), dystonic movements (HP:0001332)	6 months: infantile spasms, seizures controlled with benzoate and antiepileptics
Other symptoms	Liver dysfunction (HP:0001410), thrombocytopenia (HP:0001873)		Poor feeding (HP:0011968) requiring gastrostomy			
EEG	Burst suppression (HP:0010851)	Burst suppression (HP:0010851), paroxysmal epileptic	Hypsarrhythmia (HP:0002521) with periodic attenuation, multifocal epilepsy (HP:0031165)	No epilepsy	No epilepsy, unremarkable	Normal
Outcome	Deceased day 18	Care withdrawal and demise day 7	Deceased after brief illness age 11 months	Stable, developmental delays (DQ 55–60), tremors (HP:0001337), speech delays, (HP:0000750) hyperactivity (HP:0000752), poor short-term memory	Stable, developmental delays (DQ 70), dystonic movements (HP:0001332), truncal hypotonia (HP:0008936), limb stiffness, proximal limb muscle stiffness and hyperreflexia (HP:0002191), hyperactivity (HP:0000752), irritability (HP:0000737), single febrile seizure (HP:0011171)	17 months: Seizure free, discontinue antiepileptics, only benzoate, developmental delays 3 years: hyperactivity (HP:0000752), agitation (HP:0000713), DQ ±55 5 years: stumbles Normal exam, hyperactive (HP:0000752), autist form (HP:0000729)
Plasma glycine μM (Ref 77–376)	1406	1461	NA	715	812	1381
CSF glycine μM (Ref 2–10)	170	288	NA	65	65	88
CSF/plasma glycine ratio (Ref <0.02)	0.12	0.20	NA	0.09	0.08	0.06
Lactate (Ref 0.7–2.1)	Plasma 1.4 mm CSF 2.1 mm	Normal plasma	NA	NA	Plasma 1.3–7.0 mm CSF 1.1 mm	NA
Brain MRI	Lesions middle and anterior cerebral artery distribution, caudate, thalami	Diffusion restriction in PLIC, corticospinal tract, central tegmental tract and cerebellar white matter. Also, diffusion restriction anterior medial thalami	Linear hypointense T2 area in subcortical white matter, mostly parietal	Normal age 17 months	Day 7: diffusion restriction corticospinal tract, cerebellar, CTT, PLIC 18 months: no diffusion restriction, enhance peritrigonal; 16 months: possibly thin caudate nuclei with mild diffusion restriction	Diffusion restriction of the entire supratentorial white matter, also in middle cerebellar peduncle
Corpus callosum	Mild hypoplasia	Thin corpus callosum (HP:0033725), low normal length	Hypoplasia, thin genu and body, absent splenium and rostrum		Borderline short, normal thickness	Normal thickness and length
MRS	NA		NA	NA	Low NAA, very small glycine peak	

CTT = central tegmental tracts; PLIC = posterior limb of the internal capsule; NA = not available; NAA = N-acetyl-L-aspartate; MRS = magnetic resonance spectroscopy.

Three patients presented with an early onset severe fatal course. Patient 1 developed lethargy and hypotonia at age 12 h, which progressed to apneic coma with seizures with burst suppression on electroencephalogram (EEG). She died at age 18 days. Brain magnetic resonance imaging (MRI) showed lesions in areas related to the middle and anterior cerebral artery, and in caudate and thalamic nuclei. Patient 2 presented on day 3 with hypoglycemia and lethargy, which progressed to apneic coma with myoclonias and epilepsy and burst suppression on EEG and a pattern with paroxysmal epileptic activity, to which the child succumbed on day 7. Brain MRI studies showed thin but not short corpus callosum, diffusion restriction in white matter tracts typical for neonatal NKH (21) and diffusion restriction in the anterior medial thalami (Fig. 2A–C). Patient 3 presented at age 2 months with infantile spasms, partial seizures, hypsarrhythmia on EEG and multifocal epileptiform activity. The epilepsy gradually worsened and was never controlled, and development became more delayed, and she succumbed shortly before her first birthday. Brain MRI at age 2 months showed pronounced corpus callosum hypoplasia and hyperintense T2 signals in the subcortical white matter. She had elevated blood lactate.

Three cases with an attenuated phenotype had long-term survival. Patient 4 presented at age 6 months following an immunization with loss of skills and developmental delays but normal brain MRI. Her clinical course showed developmental delays, hyperactivity, limited communication skills, aggressive outbursts, impulsivity, defiance and occasional hallucinations, worsening during infections. Liver GCS activity was decreased 4.3 U/g protein (normal range 12–175 U/g). Benzoate treatment was discontinued for ineffectiveness. At current age 17 years, she is stable with a developmental age equivalent of 10 years, has speech delay and hyperactivity and is often emotional.

Patient 5 presented neonatally with apneic coma and diffusion restriction typical of NKH noted on brain MRI. Glycine levels were elevated and treatment with benzoate provided resumption of breathing. With continued benzoate and dextromethorphan treatment, the child has moderate global developmental delay and a dystonic movement disorder. At age 20 months, he had a 6-month global developmental delay, generalized dystonia, limited verbal communication, irritability and hyperactivity, but no seizure disorder. Brain MRI at age 18 months showed diminished volume of the caudate nuclei (Fig. 2F).

Patient 6 presented at age 3 months with left-sided partial seizures, but normal interval EEG. Brain MRI showed diffusion restriction of the entire supratentorial white matter, as described in NKH (Fig. 2D and E) (21). At age 6 months, he developed flexion spasms, unresponsive to antiepileptic treatment, but responding to benzoate treatment for elevated glycine levels, and anticonvulsant treatment was discontinued at age 1 year 5 months, as the EEG had normalized. A developmental evaluation at age 3 year 7 months showed developmental delay with a cognitive verbal level of 2 years 1 month, expressive language 1 year 4 months, receptive language 1 year 8 months and fine motor 2 years 7 months.

### Genetic findings and structural modeling of missense changes

Exome sequencing identified biallelic *GCSH* variants in the six unrelated index cases (Table 2). Compound heterozygous variants were identified in the non-consanguineous families 1, 2 and 3, and homozygous variants in the consanguineous families 4, 5 and 6. Family 1 showed the missense variant c.170A > G p.(His57Arg), and an exonic deletion c.148-?_228 +?del encompassing exon 2. Family 2 showed a paternal 1 bp insertion c.293-2_293–1insT, affecting the exon 4 acceptor splice site and a maternal variant affecting the initiator methionine c.1A > G, p.(Met1?). In family 3, the paternal allele showed the same initiator methionine change c.1A > G, p.(Met1?) in combination with a maternal nonsense variant c.226C > T, p.(Gln76^*^). Families 4 and 6 showed the homozygous missense variants c.442A > C, p.(Thr148Pro) and c.344C > T, p.(Pro115Leu) respectively. Family 5 showed a duplication c.(292 + 1_293–1)_(^*^919_?)dup encompassing the last two exons 4 and 5 of the *GCSH* gene with genomic coordinates involving positions chr16:81116399-81 118 259. In all cases, segregation analysis confirmed biallelic inheritance from the parents, except for family 4 where a copy number variant was excluded by microarray.

**Figure 3:**
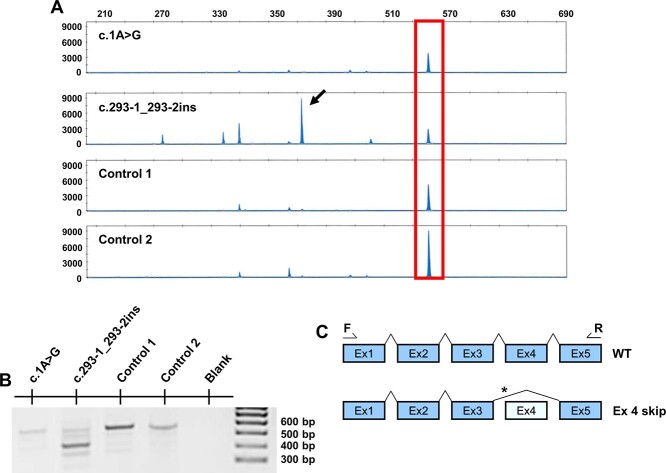
Analysis of the impact on mRNA splicing of the splice site variant in patient 2. Analysis of RT-PCR products from parental samples of patient 2 (maternal, c.1A > G and paternal, c.293–1_293-2insT) is shown along with two control samples. (**A**) Semi-quantitative capillary electrophoresis was carried out using a fluorescent reverse primer. Fragment sizes and relative fluorescent units are indicated on the x- and y-axis, respectively. Blue peaks indicate transcripts, where the wild-type (WT) transcripts (566 bp) are marked by the red box. The major alternative transcript in the paternal sample (424 bp) is shown by an arrow. (**B**) Agarose gel electrophoresis of RT-PCR products, with cDNA generated using 500 ng of RNA using a mixture of random hexamer and oligo-dT template primers, and PCR with primers located in exons 1 and 5. These shows a band of smaller size in the paternal sample compared with the controls. (**C**) Diagram showing WT and paternal major transcript, where the c.293–1_293-2ins variant is shown by an asterisk, and results in a major alternative transcript skipping exon 4 (132 bp) leading to an in-frame deletion of 44 amino acids (p.(Asp98_Asp141del).

To evaluate the variants’ effects, we used different bioinformatic analyses (Supplementary Material, Table S1) and experimental approaches. The exonic copy number changes c.148-?_228 +?del and c.(292 + 1_293–1)_(^*^919_?)dup and the nonsense variant p.(Gln76^*^) are obvious loss-of-function changes and hence likely pathogenic. According to ACMG criteria, variant c.1A > G, previously reported in three consanguineous families (20), was considered a null variant due to loss of the normal translational initiation codon and is classified as pathogenic. The c.293-2_293–1insT variant on the paternal allele of patient 2 affects the 3′-acceptor splice site of exon 4. We analyzed this by comparing the paternal sample to two controls and the maternal samples. As shown in Figure 3A, a 566 bp wild-type (WT) RT-PCR product was seen in all samples. However, the paternal sample had less WT 566 bp fragment, and showed a major 424 bp RT-PCR product (Fig. 3A). Likewise, RT-PCR with primers in exons 1 and 5 again produced a major product of lower molecular weight (MW) in the paternal sample than in controls (Fig. 3B). These results are consistent with the major alternative paternal transcript skipping exon 4 (132 bp) leading to an in-frame deletion of 44 amino acids p.(Asp98_Asp141del) (Fig. 3C), demonstrating pathogenicity of this variant. The remaining three missense variants p.(His57Arg), p.(Pro115Leu) and p.(Thr148Pro) were provisionally considered variants of uncertain significance requiring additional functional data for classification.

**Table 2:** Genetic variants and their functional impact

	Patient 1	Patient 2	Patient 3	Patient 4	Patient 5	Patient 6
Severity	Severe neonatal	Severe neonatal	Severe infantile	Attenuated	Attenuated	Attenuated
**Variants**						
Variant 1	c.170A > G	c.1A > G	c.1A > G	c.442A > C	c.(292 + 1_293–1)_(^*^919_?)dup	c.344C > T
Variant effect 1	p.**His57Arg**	p.Met1?	p.Met1?	p.**Thr148Pro**	Dup exons 4–5	p.**Pro115Leu**
Variant 2	c.148?_228?del	c.293-2_293–1insT	c.226C > T	c.442A > C	c.(292 + 1_293–1)_(^*^919_?)dup	c.344C > T
Variant effect 2	Deletion exon 2	Splicing IVS3 defect p.(Asp98_Asp141del)	p.Gln76^*^	p.**Thr148Pro**	Dup exons 4–5	p.**Pro115Leu**
**Modeling**						
H-protein stability	ΔΔG −1.22 kcal/mol			ΔΔG 0.04 kcal/mol		ΔΔG −2.89 kcal/mol
P-protein binding	ΔΔG −1.51 kcal/mol			ΔΔG 0.92 kcal/mol		ΔΔG −1.31 kcal/mol
**Functional**						
mRNA	NA	Loss of exon 4	NA	NA	NA	NA
GCS assay	NA	NA	NA	Liver GCS↓	NA	NA
***Fibs/CVS***		CVS			Fibroblasts	Fibroblasts
H-protein	NA	H-protein ↓	NA	NA	H-protein ↓	H-protein ↓
Lipoyl-proteins		Lipoylated prot.↓			Lipoylated prot.↓	Lipoylated prot.↓
PDH activity					Activity ↓ 5.2	Activity ↓ 3.6
O_2_ reserve capacity					43.6% of controls	58.5% of controls
***COS7 cells***	p.His57Arg	p.Met?	?p.Gln76^*^?	p.Thr148Pro		p.Pro115Leu
H-protein	H-protein ↑	H-protein ↓		H-protein normal		H-protein normal
Lipoyl-H-protein	Lipoylated H normal	Lipoylated H↓		Lipoylated H↓		Lipoylated H↓
Glycine exchange	GExch ↑151 ± 18.3%	GExch ↓ 28 ± 3.5%		GExch↓ 33 ± 5.3%		GExch ↓ 60 ± 7.4%
Lipoylated DLST	Lipoylated DLST↓	Lipoylated DLST↓		Lipoylated DLST↓/−		Lipoylated DLST↓
***Yeast***	Gcv3His51Arg			Gcv3Lys143Pro†		Gcv3Pro110Leu
Growth on glucose	Normal			Not informative†		Normal
Growth on glycerol	Normal			Not informative†		Normal
Growth on glycine	Decreased			Not informative†		Decreased
Respiration at 36°C	Strong ↓			Not informative†		Moderate ↓
Lipoylated Kgd2	Strong ↓			Not informative†		Moderate ↓
**Purified proteins**						
Glycine exch 1 μm	65.7 ± 5.3%^*^↓			54.0 ± 5.9%↓		87.8 ± 2.3%
Glycine exch 0.1 μm	83.4 ± 3.6%			32.4 ± 9.3%^*^↓		83.0 ± 3.7%

†variant present in a region not conserved and thus not considered an adequate model in yeast. ^*^Statistically significant result.

GExch: glycine exchange reaction.

**Figure 4:**
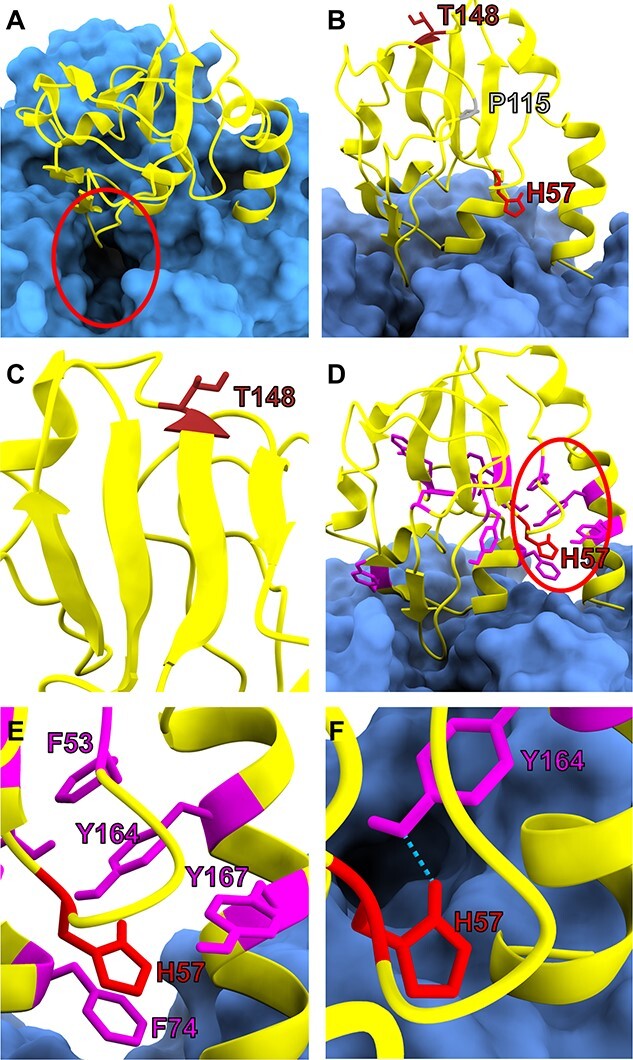
Molecular modeling of the interaction of the H-protein and the P-protein. (**A**) The predicted interaction model, with P-protein shown in blue and H-protein shown in yellow. H-protein lipoylated lysine accesses P-protein’s active site through a known tunnel from P-protein’s surface to the inner active site. The H-protein loop that contains the H-protein lipoylation site Lys107 is perched just above the active site entry tunnel in the P-protein (circled in red), as would be expected for an interaction between the two proteins. (**B**) The position of mutated residues His57 (red), Pro115 (gray) and Thr148 (brown) cannot be considered to be at the interaction interface with the P-protein as all three are not within 5 Angstroms of the P-protein’s surface. (**C**) Thr148 is part of the four-stranded β-sheet that forms H-protein’s core. The observed variant p.Thr148Pro would likely shorten the β-strand that Thr148 is in because prolines with their restricted flexibility are incompatible with β-strands. Thus, inclusion of proline at this position would likely destabilize the core β-sheet in H-protein. (**D**) His57 is positioned in an aromatic-rich region of H-protein (circled in red) with 5 of the 11 aromatic residues (shown in magenta and red) in H-protein occurring within this small region, which therefore is likely highly hydrophobic. (**E**) His57 lies close to aromatic residues Phe53, Phe74, Tyr164 and Tyr167. Introduction of the highly hydrophilic arginine in the variant His57Arg would be disruptive. (**F**) Further, His57 is predicted to form a hydrogen bond with Tyr164 which would be lost with the introduction of arginine. This region of H-protein forms a clamp which latches onto a ridge on the P-protein (A, D) and destabilization of this region through the introduction of arginine would likely interfere with the interaction between P-protein and H-protein.

To evaluate the impact on the GCS, we first modeled how these missense variants affect the H-protein structure and its interaction with the P-protein using a previously described model (22). In this predicted interaction model, the H-protein lipoylated lysine accesses the P-protein’s active site through a known tunnel from its surface to the inner active site, and the H-protein loop containing the lipoylation site Lys107 is perched just above this active site entrance tunnel of the P-protein (Fig. 4A). The mutated residues His57 and Thr148 are not located at the interaction interface with the P-protein since both are at a distance >5 Å from the P-protein’s surface (Fig. 4B). However, Thr148 is part of the four-stranded β-sheet in the H-protein’s core, and variant Thr148Pro would shorten this β-strand since the restricted flexibility of proline is incompatible with a β-strand conformation and destabilizes this core β-sheet (Fig. 4C). The His57 is located in a highly aromatic-rich region of H-protein where 5 of the 11 aromatic residues are located, including Phe53, Phe74, Tyr164 and Tyr167 (Fig. 4D and E). This creates a very hydrophobic area, which is disrupted by the introduction of the highly hydrophilic arginine in the His57Arg variant. Furthermore, His57 is predicted to form a hydrogen bond with Tyr164 which is lost with the introduction of arginine (Fig. 4F). This region of H-protein forms a clamp which latches onto a ridge on the P-protein (Fig. 4A and D), and destabilization of this region through the introduction of arginine likely interferes with the interaction between P-protein and H-protein.

We quantitatively predicted the destabilizing impact of these variants on H-protein stability using CUPSAT, which considers a ΔΔG ≤ −1.5 kcal/mol as destabilizing. Variant p.Pro115Leu was predicted to be destabilizing with ΔΔG −2.89 kcal/mol. Pro115 is part of an unstructured loop, and the change of the inflexible proline side chain to the flexible leucine appears to be deleterious to protein stability. The other two variants p.His57Arg and p.Thr148Pro did not have a significant destabilizing effect, with predicted ΔΔG of −1.22 and 0.04 kcal/mol, respectively. Variants that do not affect H-protein stability may still impact binding stability between the H-protein and the P-protein. We predicted this quantitatively using Mutabind2, which considers ΔΔG of binding ≥1.5 or ≤−1.5 kcal/mol as destabilizing. Variant p.His57Arg had a ΔΔG of binding −1.51 kcal/mol, which is predicted to be borderline. The other two variants p.Pro115Leu and p.Thr148Pro had a predicted ΔΔG of only −1.31 and −0.92 kcal/mol, respectively, and thus are predicted as not destabilizing.

### Functional analysis in patients’ cells showed impaired lipoylation and mitochondrial bioenergetic capacity

**Figure 5:**
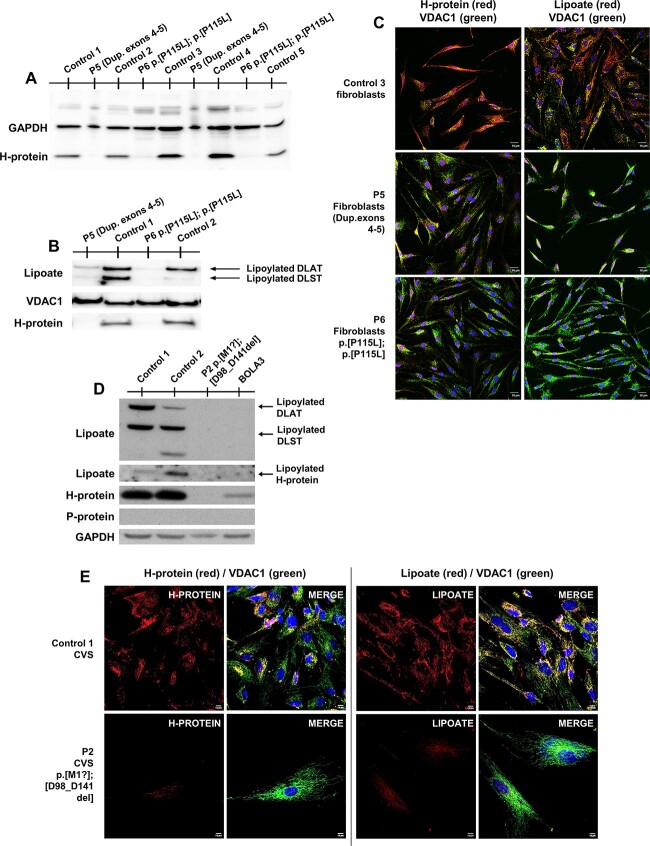
Protein studies in patient cell lines. (**A**) Patient 5 and 6 fibroblasts whole cell lysates. Western blot was stained with an antibody against GCS H-protein. GAPDH was used as loading control. (**B**) Isolated mitochondria from human primary skin fibroblasts. Western blot was stained with an antibody against lipoylated proteins. Bands represent lipoate bound to PDH E2 subunit (DLAT) and αKGDH E2 subunit (DLST). (**C**) Immunohistochemical staining using antibodies against GCS H-protein or against lipoylated proteins (red color) and VDAC1 (porin, mitochondrial marker, labelled in green). (**D**) Representative western blot of whole protein lysates from control and patient 2 CVS. GAPDH was used as loading control. CVS did not show GCS-P protein expression. BOLA3 cells present confirmed lipoylation defects and are used as negative control. (**E**) Immunofluorescence staining and confocal imaging showing different GCS H-protein expression and lipoylation patterns between control and patient 2 in chorionic villus cells (CVS).

**Figure 6:**
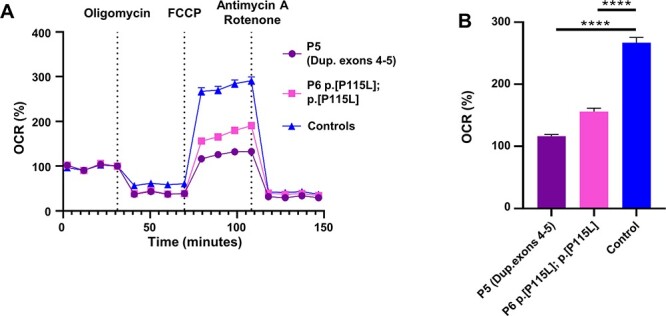
Cellular studies in patient cell lines. (**A, B**) Oxygen consumption rate (OCR) measured with XF96 extracellular flux analyzer (Seahorse) in fibroblasts of two *GCSH* individuals and controls. (A) OCR in pmol/min/norm. Unit. (Number of measurements: patient 1, *n* = 13; patient 2, *n* = 13; control 1, *n* = 13; control 2, *n* = 13; control 3, *n* = 14; control 4, *n* = 12). (B) OCR after 4 μM FCCP addition (patient 1, *n* = 13; patient 2, *n* = 13; 4 controls, *n* = 52). Bar chart shows maximal respiration. (A, B) Mean ± SEM is shown. ^*^^*^^*^^*^P < 0.0001.

Fibroblasts were only available from patients 5 and 6, and chorionic villus cells (CVS) from patient 2. These cells do not express P-protein but the H-protein and its role in lipoate synthesis can be probed. Immunohistochemical staining showed a reduced H-protein amount, and near absence of staining for lipoylated proteins in the mitochondria in all three cell lines (Fig. 5C and E). On western blot analysis, there was a strongly reduced amount of H-protein (Fig. 5A and D) and strongly reduced lipoylated proteins of the E2 subunits of PDH (DLAT) and 2-KGDH (DLST) in all cells with a small residual amount visible in patient 5 (Fig. 5B). Consequently, PDH enzyme activity determined in fibroblasts’ mitochondrial extracts showed decreased activities in patient 5 at 5.2 nmol/min^*^mg protein and patient 6 at 3.6 nmol/min^*^mg protein (normal range 6.0–19.7 nmol/min^*^mg protein). The functional impact of the reduction in bioenergetic enzymes, evaluated by measuring the oxygen consumption rate (OCR) using XF96 extracellular flux analyzer (Seahorse), showed similar baseline OCR between patients 5 and 6 and four controls, but the maximum reserve capacity was significantly reduced to 43.6% of controls in patient 5 and 58.5% in patient 6 (*P* < 0.0001) (Fig. 6A, B and E). Mitochondrial oxidative phosphorylation enzyme activities did not show significant changes compared with normal controls (Supplementary Material, Table S2). This illustrated that this decrease in lipoylation at the level of PDH and the Krebs cycle impacted the bioenergetics capacity of these cells.

### *GCSH* knockdown cell model recapitulates data from patients’ cells and shows the different effects of missense *GCSH* variants on GCS activity and protein lipoylation

**Figure 7:**
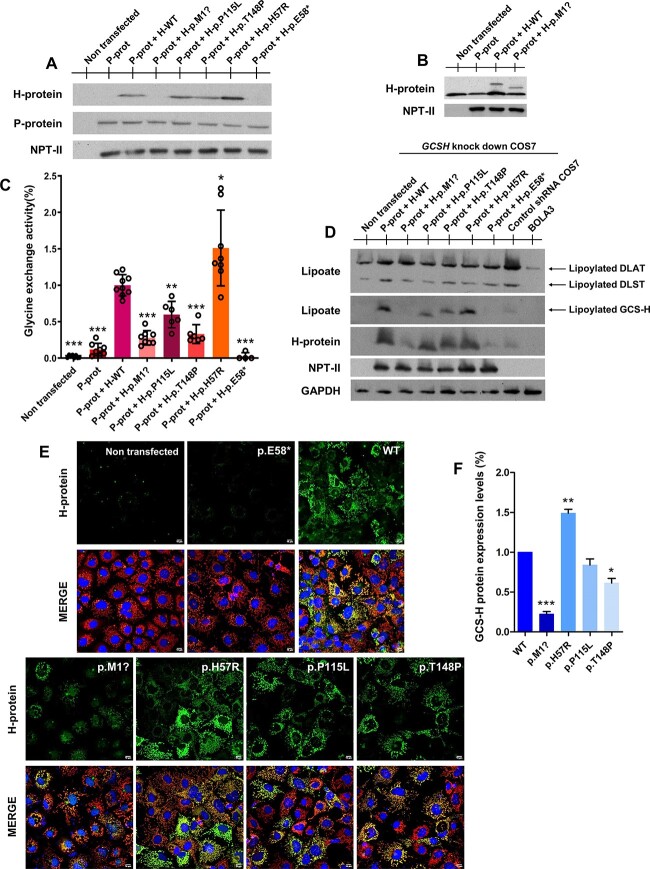
Functional effect of variants in *GCSH* expressed in a COS7 cell model. (**A**) Representative western blot of protein lysates from *GCSH* knockdown COS7 cells, co-transfected with pCMV-*GLDC* wild type (WT) and pCMV-*GCSH* WT or pCMV-*GCSH* variants and blotted with anti-GCS P-protein and anti-GCS H-protein. Neomycin phosphotransferase II (NPTII) was used as transfection control. (**B**) Representative western blot of protein lysates from control shRNA COS7 cells co-transfected with pCMV-*GLDC* WT and pCMV-*GCSH* WT or pCMV-*GCSH* p.M1? and blotted with anti-GCS-P protein and anti-GCS H-protein revealing a band of lower molecular weight for the p.Met1? variant compared with WT. (**C**) Glycine exchange enzyme activity following co-transfection of pCMV-*GLDC* and either pCMV-*GCSH* WT or pCMV-*GCSH* variant constructs into *GCSH* knockdown COS7 cells with the activity expressed as a percentage of the activity obtained for WT GCSH, which was set to 100%, with data shown from three different transfections assays measured each at least in duplicate. Differences were considered to be significant at ^*^^*^^*^P < 0.001. (**D**) Representative western blot of protein lysates from *GCSH* knockdown COS7 cells, co-transfected with *GLDC* WT and pCMV-*GCSH* WT or pCMV-*GCSH* variants and blotted with anti-lipoic and anti-GCS-H protein after 72 h. (**E**) Immunofluorescence staining and laser scanning confocal imaging of GCS-H protein. Representative merged images (40X objective lens) showing colocalization of mitochondrial marker cytochrome C (red) and GCS H-protein (green) in knockdown COS7 cells co-transfected with pCMV-*GCSH* WT or variants. (**F**) Quantification of fluorescence intensity of GCS H-proteins expressed as a percentage of the fluorescence signal obtained for WT GCSH. Differences were considered to be significant at ^*^  *P* < 0.05; ^*^^*^  *P* < 0.01; ^*^^*^^*^P < 0.001.

To study the impact of *GCSH* variants on H-protein in transfected COS7 cells, the abundance of endogenous natural H-protein needed to be reduced without causing cell death. Stable expression of short hairpin ribonucleic acids (shRNAs) following lentiviral transduction showed that TRCN0000083397 provided the strongest decrease in H-protein (to 4.9 ± 0.56% of controls (mean ± SEM)) without becoming lethal (Supplementary Material, Fig. S2A and B). On microscopy, the decrease in mitochondrial localized H-protein was accompanied by a decrease in lipoylated proteins (Supplementary Material, Fig. S2C). These cells were then transfected with various *GCSH* constructs using p.Glu58^*^ as affected control for studies of protein amount and localization (Fig. 7A, D and E), and co-transfected with WT *GLDC* to evaluate the glycine exchange reaction (Fig. 7C). Transfection with WT H-protein increased lipoylation of both DLAT and DLST maximally at 72 h. In comparison, transfection with p.Glu58^*^ showed markedly less lipoylation recovery for DLST (Fig. 7D), whereas the impact on DLAT lipoylation was less clear, possibly due to the remaining small amount of endogenous lipoylated H-protein.

In this new expression system, the p.Met1? variant resulted in a near absent amount of normal sized H-protein (Fig. 7A) and lipoylated H-protein (Fig. 7D), with decreased mitochondrial localization (Fig. 7E and F). Transient transfections of p.Met1? in non-target shRNA transduced COS7 cells revealed a smaller sized H-protein (Fig. 7B)**,** and the glycine exchange activity decreased to 25 ± 3.5% of WT but was not absent as observed for p.Glu58^*^ (Fig. 7C). Furthermore, the lipoylation of DLST was markedly less than that of WT H-protein and similar to the null variant p.Glu58^*^ (Fig. 7D).

The p.Pro115Leu variant showed an almost normal amount of H-protein correctly located in mitochondria (Fig. 7A, E and F). The glycine exchange reaction was reduced to 60 ± 7.4% of controls (Fig. 7C). There was a decrease in the amount of lipoylated H-protein and a reduced lipoylation of DLST compared with WT (Fig. 7D). The p.Thr148Pro showed a mildly reduced presence of H-protein, which was also mitochondrially localized (Fig. 7E and F). The glycine exchange activity was decreased to 33 ± 5.3% (Fig. 7C). The lipoylated H-protein amount was decreased. DLST lipoylation was also reduced, although less than for p.Pro115Leu (Fig. 7D).

Surprisingly, the p.His57Arg variant showed an increased level of H-protein on western blot and mitochondrial colocalization with an increased glycine exchange activity to 151 ± 18.3% over WT control (Fig. 7A, C, E and F). Although the lipoyl-H-protein amount was similar to controls, the lipoylated DLST levels and even DLAT levels did not recover, reflecting a decrease in lipoyl transfer (Fig. 7D). The results in this model system illustrated a dissociation between its role in E2 lipoylation and GCS activity.

### Changes p.His57Arg and p.Pro115Leu impair mitochondrial respiration and cell growth in yeast

**Figure 8:**
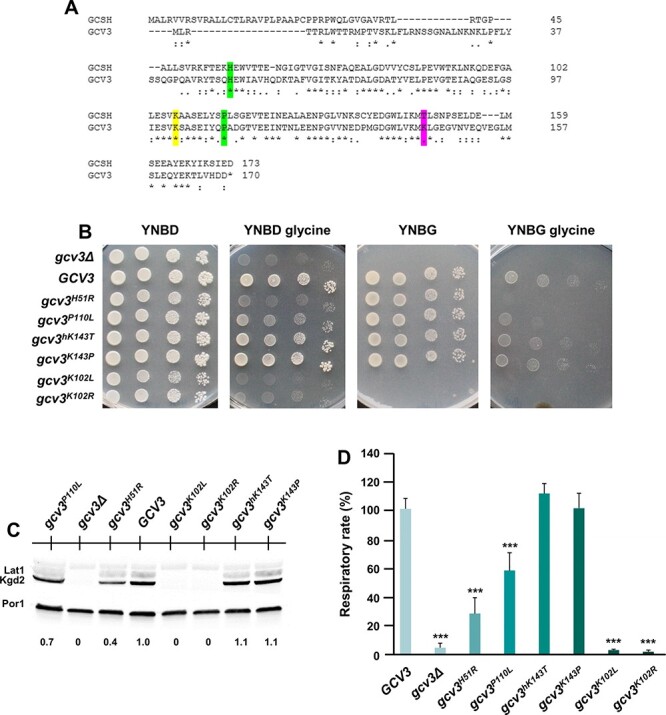
Yeast model of missense variants in the homologous gene *GCV3*. (**A**) Alignment (Clustal Omega) of the human GCSH and the yeast Gcv3 proteins. The studied residues are highlighted in green for conserved amino acids and in magenta for non-conserved amino acids. Lysine covalently modified by lipoic acid is indicated in yellow. Amino acids are indicated as conserved (^*^), with strongly similar properties (:) or with weakly similar properties (.). (**B**) Growth test: the *gcv3Δ* strain harboring wild type, humanized or mutant alleles or the empty vector were serially diluted and spotted on YNB agar plates supplemented with the fermentable carbon source glucose (YNBD) or the non-fermentable carbon source glycerol (YNBG), or on these media using glycine as the sole nitrogen source (YNBglycine) and incubated at 28°C. (**C**) Representative western blot on total protein extract using an anti-lipoic acid antibody visualizing the lipoylation of Lat1 and Kgd2, and anti-Por1 as loading control. The Kgd2 quantification was performed on two independent blots using Image Lab Software (Bio-Rad). (**D**) Respiratory activity: cells were grown at 36°C in SC medium containing glycine as nitrogen source supplemented with 0.6% glucose. Values were normalized to the *GCV3* wild-type strain. The data are the results of at least three measurements, and the error bars indicate the standard deviation. Statistical analysis was performed comparing mutant strains to wild type using a two-tail unpaired Student’s t test: ^*^^*^^*^  *P* < 0.001; ns: not significant.

The variants identified in human *GCSH* were also modeled using the yeast *Saccharomyces cerevisiae* as a system taking advantage of the orthologous gene *GCV3*. As shown by protein alignment (Fig. 8A), the human residues His57 and Pro115 are conserved between yeast and human, corresponding to yeast His51 and Pro110, respectively, and mutant alleles *gcv3^H51R^* and *gcv3^P110L^* were created. In yeast, Lys102 is covalently lipoylated, which is required for lipoylation of Lat1 (DLAT equivalent in PDH) and Kgd2 (DSLT equivalent in 2-KGDH). As reference strains *gcv3^K102R^* and *gcv3^K02L^* were constructed; carrying a mutation makes the residue inactive as lipoate acceptor (4). Lipoylated Gcv3, and not glycine cleavage activity *per se*, is responsible for lipoylation of Lat1 and Kgd2 (4). The WT and the mutant alleles, cloned into a centromeric vector under the control of the endogenous promoter region, were introduced into a yeast strain deleted of the *GCV3* gene.

Under fermentable media YNBD, all strains grew equally well, whereas in non-fermentable media YNBG containing glycerol, the null mutant *gcv3Δ* and mutants affecting Lys102 were severely affected, but the missense variants under study were unaffected (Fig. 8B). Gcv3 is involved in glycine cleavage and in yeast it is essential for growth when glycine serves as the sole nitrogen source (23). Absent growth occurred for the mutant controls *gcv3Δ*, *gcv3^K102R^* and *gcv3^K102L^* using glycine as nitrogen source. In this setting, the growth of *gcv3^H51R^* was severely limited, and that of *gcv3^P110L^* moderately limited indicating a problem in glycine cleavage activity of GCS. The *gcv3Δ*, *gcv3^K102R^* and *gcv3^K102L^* showed as expected near absent lipoylation of Kgd2 (Fig. 8C). The strain *gcv3^H51R^* showed strongly reduced Kgd2 lipoylation, and the strain *gcv3^P110L^* showed moderately reduced lipoylation. As expected for this effect on key bioenergetic enzymes, the respiratory activity at 36°C in SC medium containing glycine for *gcv3^H51R^* was severely reduced (28 ± 11% residual activity relative to control), and for *gcv3^P110L^* moderately reduced (58 ± 12% activity relative to control), whereas the affected controls *gcv3Δ*, *gcv3^K102R^* and *gcv3^K102L^* (5 ± 3% activity) were sharply reduced (Fig. 8D). Thus, these experiments indicate a strong impact of these variants on glycine cleavage activity of the GCS and on lipoylation with *gcv3^H51R^* more severe than *gcv3^P110L^*. The human amino acid residue Thr148 is not conserved in yeast, or across multiple fungi species, corresponding to Lys143. Both the humanized version *gcv3^hK143T^* and the potentially pathogenic variant *gcv3^K143P^* were made. However, they did not show a functional effect in any assay. This likely did not represent an adequate model, possibly due to an evolutionary changed role of this residue.

### Purified recombinant human proteins demonstrates a primarily impact of p.Thr148Pro on GCS activity

WT H-protein and variants p.His57Arg, p.Pro115Leu and p.Thr148Pro were expressed in *Escherichia coli* and then purified. They were all similarly lipoylated by LplA to form lipoylated H-protein (including each of the variants) and purified with a small amount of LplA and an unidentified unlipoylated impurity of approximately 25 kDa remaining. This was used to evaluate the interaction of the H-protein with the P-protein for the impact on the GCS function as assessed in the glycine exchange reaction. GCS activity was most significantly reduced to 32.4 ± 9.3% and 54.0 ± 5.9% of WT for p.Thr148Pro at 0.1 and 1 μM H-protein, respectively, less so for p.Pro115Leu to 83.0 ± 3.7% and 87.8 ± 2.3% and for p.His57Arg to 83.4 ± 3.6% and 65.7 ± 5.3% at 0.1 and 1 μM (Supplementary Material, Fig. S3A and B).

## Discussion

We describe six children with biallelic pathogenic variants in *GCSH*. The clinical presentations observed can be categorized into severe, neonatal or infantile presentations (cases 1–3), and more attenuated presentations (cases 4–6). Based on the dual role of H-protein in lipoylation of bioenergetic enzymes PDH and 2-KGDH, and its direct involvement in the GCS (Fig. 1), we would expect these pathogenic variants to result in clinical and biochemical symptoms generally similar to both PDH deficiency and NKH. Shared features of these conditions of developmental delays and seizures occurred in most patients. As in NKH, all patients had elevated CSF glycine levels, higher in severe than in the attenuated cases (Supplementary Material, Fig. S1). Three patients (1,2,5) had a neonatal episode typical for NKH with coma, respiratory insufficiency, myoclonia and burst suppression on EEG. Three patients (4–6) had a phenotype typical of attenuated classic NKH, intermediate to good subtype, of developmental delays with hyperactivity, and behavioral problems. Brain MRI showed the typical diffusion restriction pattern of NKH in patients 2, 5 and 6 (21). There were also symptoms related to PDH deficiency, such as movement disorders of dystonia (patient 5) and stumbling (patient 6) which are common for PDH deficiency (24–26) and not for NKH, which rather exhibits chorea (12,13,15). Lactic acidosis was rarely present (patient 5 only). A thin corpus callosum is a shared brain imaging feature of PDH deficiency and NKH (patients 1 and 2), but imaging findings typical of PDH deficiency and not seen in NKH deficiency (21,24,25,27) included a thalamic lesion (patient 2), hypoplastic corpus callosum with absence of splenium and rostrum (patient 3), subcortical white matter involvement (patient 3), and a stroke-like pattern with basal ganglia involvement (patient 1), which is a recently described destructive brain MRI presentation of PDH deficiency (27). Thus, the clinical phenotype of *GCSH* deficiency includes a combination of symptoms related to dysfunction of both GCS and PDH deficiency, albeit to a variable degree. We next relate this to the biochemical impact of the molecular causes.

Multiple lines of evidence supported the pathogenicity of the identified variants as summarized in Table 2. The pathogenic impact of an exonic deletion in patient 1 and of a nonsense variant in patient 3 are evident. For patient 2, analysis of the impact of the intronic variant on splicing showed a skip of exon 4. Such hypomorphic variants with decreased H-protein are expected to affect both GCS activity and enzyme lipoylation. Direct evidence from patient biopsy materials was only available for four patients. Three patients (2,5,6) showed decreased H-protein and lipoyl-H-protein amounts, which affected the lipoylation of E2 proteins. Only for patient 4 was a liver biopsy available to show deficient GCS activity. For comparison, the nonsense variant p.Glu58^*^ used in the COS7 study affected both functions.

The study further focused on the impact of single nucleotide variants. The p.Met1? translational initiation codon variant was found in two patients in this study and in three previously published patients (20,28), with a carrier incidence in gnomAD of 6.4 × 10^−5^. This variant resulted in greatly reduced, but not absent, H-protein, mostly of a lower MW in transfected COS7 cells. The alternative start site is not clear since the *GCSH* sequence does not contain an in-frame ATG codon until near the end at positions 147 and 159 of the 173-codon cDNA. However, the rarely used alternative start codon CTG is present in-frame at positions 12, 16, 41 and 47 (29), but direct evidence of its use is lacking. A distal start site might impact the leader peptide and affect mitochondrial localization. This hypomorphic variant caused decreased GCS activity and lipoylation of E2 proteins in COS7 cells, but still maintained some residual activity, allowing for a viable homozygous presentation, although the clinical presentation was severe.

The missense variants had variable effects. On molecular modeling, while each variant would be predicted to destabilize an important structural region, quantitatively, p.Pro115Leu had the strongest destabilizing effect. Indeed, the H-protein amount was almost undetectable in the patient 5 fibroblasts (see Fig. 5A and C) and clearly decreased in the COS7 model (Fig. 7D). The reduced lipoylated H-protein affected the lipoylation of PDH and 2-KGDH in both systems. The reduced bioenergetic capacity was reflected in reduced oxygen consumption in patient fibroblasts and in the yeast model. Molecular modeling predicted less impact on P-protein binding and indeed the mutant H-protein had *in vitro* only limited effect on the glycine exchange reaction, which in COS7 cells was only reduced by 40%. This reduction still limited growth of the yeast model on media that required GCS for nitrogen sourcing, and the child had elevated glycine levels and a phenotype primarily consistent with attenuated NKH. Thus, this hypomorphic missense variant with reduced protein stability affected both glycine metabolism and lipoylation. A complete absence of H-protein is likely not viable.

The variant p.Thr148Pro had on molecular modeling only a limited predicted impact on protein stability. Agreeing with these results, the protein amount in COS7 cells was fairly preserved, and the lipoylation, although somewhat reduced, was also the least affected of all variants. This recombinant mutant H-protein had the strongest reduction of the glycine exchange reaction *in vitro*. Also, COS7 cells exhibited the strongest reduction in the glycine exchange reaction. The clinical phenotype is consistent with attenuated NKH without a clear symptom of a bioenergetic defect. Thus, this variant appears to primarily affect glycine metabolism, with limited impact on E2-protein lipoylation.

Finally, the p.His57Arg variant showed an increased amount of protein in COS7 cells. The impact on the glycine exchange reaction was limited for purified proteins and was increased in COS7 cells. *In vivo*, this mutant still limited growth of yeast cells using glycine as the nitrogen source, and the patient’s elevated glycine levels implied an impact on glycine metabolism. However, despite near normal lipoyl-H-protein present, the marked decrease in the lipoylation of E2 proteins in COS7 cells implied decreased transfer. In yeast, this was reflected in decreased respiratory activity. Clinically, the stroke-like episode with basal ganglia involvement could represent PDH deficiency (24). This variant appeared to impact the function of H-protein as a lipoyl-donor more than glycine metabolism, although the latter was still somewhat affected.

Our study illustrates that *GCSH* variants can affect the two predicted roles of the H-protein, glycine cleavage activity and protein lipoylation (30), in three possible ways. Most common was a reduction in the amount of H-protein, which affected both functions of glycine metabolism and lipoylation. This mechanism was noted in exonic copy number variants, the splice site variant, the initiator variant p.Met1? and the missense variant p.Pro115Leu. All patients retained some protein, as complete absence of H-protein is likely not viable, as has been shown in a mouse model (31). Selected missense variants retained protein stability and affected one function more than the other. The variant p.Thr148Pro appeared to have a greater effect on glycine metabolism with only limited effect on protein lipoylation. The variant p.His57Arg had even preserved protein stability and limited impact on glycine metabolism, but did have a clear impact on lipoylation of E2 proteins. The clinical features of these two patients mimicked this preferential impact. Treatment will need to consider the possible differential functional impacts. For variants with decreased protein, treatment that improves protein expression or stability, or gene addition may be sought. For variants that affect glycine metabolism, treatments used in NKH may be helpful. Glycine reduction strategy using sodium benzoate treatment improved seizure control of infantile spasms in patient 6 and provided resumption of breathing and improved alertness in patient 5. For disorders affecting lipoylation, treatment restoring lipoylation or providing an alternative substrate for these combined deficiencies of PDH and 2-KGDH may be helpful.

The requirement for residual activity may explain the rarity of *GCSH* variants as a cause of disease and of NKH. Identifying only six patients with *GCSH* pathogenic variants, despite molecular information on over 600 NKH subjects (18), puts the incidence of pathogenic *GCSH* variants as a cause of NKH at less than 1%.

Our study has limitations. The clinical descriptions were limited by the retrospective nature, with limited access to biopsied patient materials. Our study only examined the interaction with the P-protein through the glycine exchange reaction, and not the interaction with T-protein, or a direct study of its interaction with the lipoyltransferase LIPT2. Variants could also affect the reaction of the swinging lipoyl-arm to protect the amino-methyl group, for which Ser67 is important (32).

In summary, our study identifies variants in *GCSH* as a rare cause of a combined disorder of glycine metabolism and a bioenergetic disorder due to failure of enzyme lipoylation. It joins the genetic causes of lipoate deficiency syndromes which involve lipoate biosynthetic enzymes (*LIPT2*, *LIAS*, *LIPT1*), iron–sulfur cluster biogenesis genes (*BOLA3, NFU1, GLRX5, ISCA2, IBA57)* and a disorder of intramitochondrial S-adenosylmethionine transport (*SLC25A26*) (12,30,33). Most pathogenic variants in *GCSH* result in hypomorphic alleles with reduced H-protein resulting in combined deficiency of both functions, while rare missense variants can affect one function more than the other. Clinical findings consist of symptoms from both NKH and a bioenergetic disorder similar to PDH deficiency. Our findings here illustrate the complex interplay of functional and clinical impact when variants affect a multifunctional protein, best assessed through a multi-modal investigation.

## Materials and Methods

### Clinical studies

The medical records of patients with variants in *GCSH* were reviewed. When available, brain MRI studies were reviewed by a neuroradiologist (NS). All studies were done after informed consent was obtained from all living subjects or their legal guardian. Studies in North America were done according to an institutional review board (IRB)-approved protocol (COMIRB# 05–0790), and in Madrid according to a protocol approved by the Ethics Committee of Universidad Autónoma de Madrid (CEI-105-2052). Per institutional rules, no further ethics committee review was required for other European centers. Consent for publication was received from all families.

### Genetic studies

Genetic studies were performed as part of the diagnostic workflow of each clinical laboratory. DNA was extracted from blood samples or chorionic villus cells. Next generation sequencing of affected individuals was based on customized exome panels (for families 1 and 6), Sanger sequencing (for family 4) or whole exome sequencing (for families 2, 3 and 5). Copy number variations were analyzed by multiplex ligation-dependent probe amplification (family 1) or DNA microarray assay (family 4).

Variants were filtered based on call quality, allele frequency in population databases and impact on gene function. Different inheritance models were considered. Pathogenicity predictions from CADD (34), GERP (35), PolyPhen-2 (36), SIFT (37) and Mutation Taster (38) were used to evaluate the variants’ deleteriousness. A combination of in-house analysis with data obtained from Alamut Visual version 2.15 (SOPHIA GENETICS, Lausanne, Switzerland) and the Varsome platform (39) was used to classify variants according to ACMG guidelines (40,41). Variants in *GCSH* were designated according to GenBank: NM_004483.5, and annotated with the Human Genome Variation Society recommendations by using Mutalyzer.

To evaluate the impact of the c.293-2_293–1insT variant in patient 2, paternal RT-PCR products were assessed qualitatively and semi-quantitatively and compared against two controls and a maternal sample. A total of 500 ng RNA derived from blood was retrotranscribed using a mixture of random hexamer and oligo-dT template primers, and the SuperScript IV First-Strand Synthesis System (ThermoFisher, Waltham, MA). PCR was carried out using primers in exon 1 (5′-CCTCCGCGCCTCCATCCAGCCGGC-3′) and exon 5 (5′-TACTCAGTGTCATCTTGATCAGCC-3′). The RT-PCR products were qualitatively assessed by agarose gel electrophoresis and purified using the QIAquick Gel Extraction kit (Qiagen, Germantown, MD). Products were sequenced using BigDye Terminator v3.1 Cycle Sequencing Kit on an ABI3730 Genetic Analyzer and analyzed by Sequencing Analysis v7.0 software (all Applied Biosystems, San Francisco, CA). Furthermore, the products were quantitatively assessed by capillary electrophoresis of fluorescent amplicons (ABI3730) to measure the relative abundance of each transcript detected. RT-PCR assays for semi-quantitative purposes were performed in duplicate. GeneMapper software (Applied Biosystems) was used for data visualization and peak size calling.

Finally, we used *in silico* molecular modeling to evaluate the structural effect of the identified missense variants as described (22). Human homology models were generated using SWISS model (Swiss Institute of Bioinformatics) (42–44) for P-protein using as the template the solved crystal structure for Synechocystis sp. PCC 6833 P-protein holoenzyme (PDB = 4lhc) (45) and for H-protein as template bovine H-protein crystal structure (PDB ID = 3wdn) (46). The H- and P-proteins interaction model was generated using ClusPro 2.0 via a Fast Fourier transform algorithm (47,48), and ranking the models based on three parameters: (i) evolutionary conservation of the H-interface, (ii) evolutionary conservation of the P-interface and (iii) distance between H-protein active site lysine and the entry to the P-protein active site access tunnel (22). The effect of the *GCSH* variants on its stability was predicted using CUPSAT, utilizing protein structure to make fast and accurate ΔΔG predictions from environment-specific atomic and torsion angle potentials (49). Variants with ΔΔG ≤ −1.5 kcal/mol were considered destabilizing, based on the observation that destabilizing variants in core residues have a mean ΔΔG of ~ −1.4 kcal/mol (50). The effects of variants on stability of the interaction between the H-protein and P-protein were predicted using Mutabind2, which predicts ΔΔG of binding affinity using statistical potentials, molecular mechanics force fields and fast side-chain optimization algorithms built via the random forest method (51). Mutabind2 defines deleterious variants as those having a ΔΔG ≥1.5 or ≤−1.5 kcal/mol. Destabilizing ΔΔG’s are represented as negative values, while stabilizing ΔΔG’s are represented as positive values.

### Patient cell studies

#### Determination of the oxygen consumption rate by Seahorse XFe96 analyzer

Primary human skin fibroblasts of affected individuals and controls were cultured in DMEM with 10% fetal bovine serum to 80% confluence, prior to seeding 12 000 cells in 80 μl cell suspension in a 96-well plate and incubating overnight. In a Seahorse XFe96 analyzer (Agilent Technologies, Santa Clara, CA), after overnight calibration of the sensor cartridge with water at 37°C in ambient air condition, it was incubated with 200 μl XF Calibrant pH 7.4 at 37°C without CO_2_. For the assay, Seahorse XF Media pH 7.4 was supplemented with 2 mm L-glutamine, 10 mm glucose and 1 mm sodium pyruvate, and 180 μl was added to the cells, and the plate was incubated at 37°C without CO_2_ for 1 h. During the assay, 20 μl of oligomycin (50 μm), 22 μl of FCCP (40 μm) and 25 μl antimycin A (5 μm) together with rotenone (5 μm) were injected into the ports, and after each injection, four measuring points were recorded.

#### Enzyme activities in isolated mitochondria from fibroblasts

Mitochondria extracts isolated from fibroblasts according to (52) were used to measure enzyme activities, all performed in duplicate. Oxidative phosphorylation enzyme activities of complexes I through V were determined spectrophotometrically at 37°C (Uvicon 922, Kontron, Milan, Italy) as described (52,53). PDH complex was measured by a radiochemical assay as described (54) by using 2 μl of fibroblast mitochondria per assay in a total reaction volume of 100 μl. The normal range was established from 22 controls.

#### Lipoic acid immunohistochemical staining and western blot

Primary human skin fibroblasts were seeded on chamber slides at moderate confluence. They were fixated overnight in 4% formalin at 4°C, then washed with phosphate buffered saline (PBS) containing 0.5% Tween-20 (PBS-T). Antigen retrieval was performed for 40 min at 95°C with 1 mm EDTA buffer containing 0.05% Tween. After cooling and washing, the proteins were detected with primary antibodies against protein-bound lipoic acid (lipoate antibody), VDAC1, in DAKO diluent (for details on all antibodies, see Supplementary Material, Table S3). These were detected with secondary antibodies Alexa Fluor 488 donkey anti-mouse and Alexa Fluor 594 donkey anti-rabbit, incubated with DAPI (1:2000), and, after washing, mounted with DAKO fluorescence mounting media and images taken with a Zeiss confocal laser microscope at 200× magnification.

Whole-cell lysates of mitochondria by differential centrifugation in 1.5 M sucrose and resuspended in SEKT buffer (250 mmol/l sucrose, 2 mmol/l EGTA, 40 mmol/l KCl, 20 mmol/l KCl [pH 7.4]) were analyzed on a 10% polyacrylamide gel electrophoresis (PAGE) before western blotting and detection with primary antibodies against H-protein, lipoate, VDAC1 and GAPDH, and detected with secondary horseradish peroxidase labeled antibodies 1:100 (EnVision kit, Dako), with the Lumi-Light PLUSPOD substrate (Roche).

### Mammalian cell studies

To reduce interference from endogenous H-protein, knockdown COS7 cells were generated using shRNA prior to transfection with mutant *GCSH* plasmids and evaluation of enzyme activities and protein functions.

#### GCSH knockdown model in COS7 cells

Lentiviral particles were generated in HEK293T by co-transfection of packing (pCMV-dR8.74) and envelope (pMD2.G) plasmids (Addgene, Cambridge, MA) with pLKO.1 plasmids containing *GCSH* shRNAs (TRCN0000083393, TRCN0000083394, TRCN0000083395, TRCN0000083396, TRCN0000083397) targeting different sequences of the human *GCSH* (NM_004483.5)*,* or a non-target shRNA (SHC002 MISSION® pLKO.1-puro Non-Mammalian shRNA Control Plasmid DNA), and were used to transduce COS7 cells, followed by puromycin selection for stable expression of the shRNA. Cell strains included control COS7 cells, non-target shRNA transduced cells and shRNA *GCSH* knockdown cells.

#### Recombinant plasmids and transient transfections

The following variants in *GCSH* were created from the mammalian plasmid pCMV6-XL5-*GCSH* (OriGene Technologies, Rockville, MD), containing the full-length open reading frame of human *GCSH*, using site-directed mutagenesis by the QuickChange Lightning site-directed mutagenesis kit (Agilent, 210 518): c.1A > G, p.Met1?; c.170 A > G, p.His57Arg; c.344 C > T, p.Pro115Leu; and c.442 A > C, p.Thr148Pro; and c.172G > T, p.Glu58^*^ as an affected control. Plasmids were validated by Sanger sequencing. These pCMV-*GCSH* constructs were co-transfected with a pCMV-*GLDC* (NM_000170.3) plasmid containing human P-protein and neomycin phosphotransferase II (NPTII) (EX-Y2609-M02; Genecopoeia, Rockville, MD) into non-target shRNA transduced COS7 and *GCSH* knockdown COS7 cells, with harvesting after 48 or 72 h. Transfection efficiency was determined by NPTII expression.

#### Enzyme activity and protein levels

P-protein enzymatic activity was determined in triplicate using the exchange reaction between radiolabeled bicarbonate NaH^14^CO_3_ and glycine as described (55), with protein levels measured by the Lowry method (56). Untransfected cells and cells transfected only with pCMV-*GLDC* WT plasmid were used as controls. After cell lysis using the lysis buffer (2% Triton X-100, 10% glycerol, 150 mm NaCl, 10 mm Tris–HCl pH 7.5, 150 mm NaCl) and freeze-thawing, supernatants were used for western blotting, with protein content measured using the Bradford method (Bio-Rad Laboratories, Hercules, CA). Components of the GCS complex were separated on 10% PAGE gels, western blotted to a nitrocellulose membrane and detected with antibodies against H-protein, P-protein and neomycin phosphotransferase II proteins using vinculin as loading control. For protein lipoylation, samples were separated on a 4–12% PAGE gel (NuPAGE™ Novex™ 4–12% Bis-Tris Protein Gels) before western blotting and detection with an anti-lipoate antibody. For all studies, a secondary horseradish peroxidase conjugated antibody was used, followed by membrane development with ECL (GE Healthcare, Piscataway, NJ), and band intensity quantified using a BioRad G-8000 scanner.

#### Subcellular localization by confocal microscopy

COS7 cells were cultured on 12 mm glass cover slips, and co-transfected with pCMV-*GLDC* and mutant pCMV6-XL5-*GCSH* constructs as above. Cells were fixed with 10% formalin (Sigma-Aldrich), permeabilized with 0.1% Triton X-100 in PBS and, after applying blocking solution (5% BSA in 0.1% Triton X-100, PBS), were incubated overnight with primary antibodies for H-protein, lipoylproteins and cytochrome c, followed by incubation with 1:500 Alexa 488 anti-rabbit and 1:500 Alexa 555 anti-mouse secondary antibodies. Visualization was performed using a LSM 710 confocal microscope coupled to Axiovertical microscopy AxioImager.M2 (Zeiss, Jena, Germany). Image analysis and fluorescence quantification were performed using Fiji (ImageJ) software.

### Yeast studies

#### Strains and culture media

The yeast strain used in this study was BY4741 *gcv3:kanMX4* (*MATa*; *his3Δ1 leu2Δ0 met15Δ0 ura3Δ0 gcv3::kanMX4*) transformed with the empty vector pFL38 or the vector carrying the WT allele or the mutant alleles of *GCV3*, the yeast homolog of *GCSH*. *GCV3* was amplified and cloned under its natural promoter in the centromeric vector pFL38 (57). *GCV3* was mutagenized by a PCR overlap technique to obtain mutant alleles (58), and cloned into the pFL38 vector. The plasmid (empty, WT or mutant allele) was introduced in the BY4741 *gcv3∆* strain using the LiAc-ssDNA-PEG method (58). Transformation was performed after growth in YPAD medium (59). The following media were used: YNB (0.69% YNB without amino acids and 0.5% ammonium sulfate), YNBglycine (YNB with 250 mm glycine replacing ammonium sulfate) which provided glycine as sole nitrogen source, SC (YNB added of 1 g/l dropout mix without uracil) (60) or SCglycine (SC with 250 mm glycine instead of ammonium sulfate). Amino acids histidine, leucine and methionine were supplemented as needed, and 20 g/l agar was used for solidification (Formedium™, UK).

#### Functional analysis and protein quantification

For growth analyses, the strains were serially diluted, spotted and grown at 28°C on YNB or YNBglycine agar plates supplemented with 2% glucose (YNBD) or 2% glycerol (YNBG). To evaluate mitochondrial respiratory activity in yeast, oxygen consumption rate was measured using a Clark-type oxygen electrode (Oxygraph System, Hansatech Instruments, UK) at 30°C with 1 ml of air-saturated respiration buffer (0.1 M phthalate–KOH pH 5.0, 0.5% glucose) from yeast cell suspensions that had been cultured for 24 h at 36°C in liquid SCglycine medium supplemented with 0.6% glucose until exhaustion. For protein analysis, yeast cells were grown as for respiratory activity analyses (24 h at 36°C in liquid SCglycine medium supplemented with 0.6% glucose until exhaustion). Protein extraction was performed with the trichloroacetic acid method, and proteins resuspended in Laemmli sample buffer pH 6.8, separated on 4–20% precast gels (Bio-Rad, USA) and western blotted on nitrocellulose filters. Proteins were detected with primary antibodies for lipoate and Por-1, followed by appropriate fluorescent secondary antibodies (anti-rabbit StarBright™ Blue520 and anti-mouse StarBright™ Blue700), with signals detected using the Chemidoc MP Imaging System and quantified with Image Lab software (Bio-Rad).

### Recombinant protein studies

Recombinant human proteins involved in the GCS and lipoyltransferase reactions were generated *in vitro* including the mutant H-proteins and evaluated for their activity in the enzymatic glycine exchange reaction and lipoyltransferase reaction.

#### Generation of recombinant proteins

DNA corresponding to the mature forms of *GCSH* and codon-optimized *GLDC* (Life Technologies, Carlsbad, CA) was each cloned into the pET-47b (+) bacterial expression vector at the *XmaI* and *PacI* sites. The missense variants p.H57R, p.P115L and p.T148P were introduced into the *GCSH* gene by site-directed mutagenesis using the QuickChange II XL mutagenesis kit (Agilent). P-proteins were expressed in Rosetta 2(DE3) (EMD Millipore) using TYP media with 0.1 mg/ml pyridoxal phosphate and 0.1 mg/ml glycine added. H-protein was expressed in BL21 Star™ *E. coli* (ThermoFisher) expression strains, grown in M9ZB minimal media to minimize the possibility of lipoylation by endogenous enzymes, and expression induced by IPTG. After opening the cells, all proteins were purified by affinity chromatography on Ni-NTA resin, followed by ion exchange chromatography step on Q-sepharose resin (GE Healthcare). Purity was assessed using SDS-PAGE. To lipoylate the purified recombinant H-proteins, lipoate-protein ligase (*LplA*) from *E. coli* was expressed, purified and used to lipoylate purified human H-proteins as described, using a molar ratio of 0.5:1.0 LplA:GCSH (16,61). The H-proteins were purified from the LplA and excess lipoate using anion exchange chromatography on Q-sepharose resin (GE Healthcare). Lipoylation was confirmed by western blot analysis using an antibody specific for protein-bound lipoate.

#### Enzymatic assays

The impact of mutant H-proteins on the GCS enzyme activity was assayed by measuring the glycine exchange activity as described in triplicate with the mutant H-protein activities expressed as percentages of WT samples measured in the same experiment (16,55,61).

### Statistics

Descriptive statistics were used to summarize data presented. Comparisons were done by Student t-test as indicated. A *P*-value of 0.05 was considered significant.

## Supplemental data

Supplemental data contains detailed case histories, three figures, and three tables containing a systematic *in silico* evaluation of the variants, mitochondrial oxidative phosphorylation and PDH enzyme activities measured in fibroblasts, and a list of antibodies used.

## Web resources

GERP: https://genome.ucsc.edu/cgi-bin/hgTrackUi?db=hg19&g=allHg19RS_BW

CADD: https://cadd.gs.washington.edu

Polyphen-2: http://genetics.bwh.harvard.edu/pph2

SIFT: https://sift.bii.a-star.edu.sg

Mutation Taster: www.mutationtaster.org

Alamut Visual version 2.15: https://www.interactive-biosoftware.com/alamut-visual/features/

Varsome platform: https://varsome.com/

Mutalyzer: https://mutalyzer.nl/

ClusPro 2.0: https://cluspro.org/login.php

Cupsat: http://cupsat.tu-bs.de

Mutabind2: https://lilab.jysw.suda.edu.cn/research/mutabind2/

gnomAD: https://gnomad.broadinstitute.org/

Human Phenotype Ontology: https://hpo.jax.org/app/

## Supplementary Material

Supplemental_information_FINAL_ddac246
